# Revealing the Most Vulnerable Groups: Courtesy Stigma in Caregivers of Autistic Persons in Quebec

**DOI:** 10.3389/fpsyg.2024.1320816

**Published:** 2024-07-31

**Authors:** Alena Valderrama, Béatrice Nikièma, Baudouin Forgeot d’Arc, Lucila Guerrero, Mathieu Giroux

**Affiliations:** ^1^Department of Social and Preventive Medicine, School of Public Health, Université de Montréal, Montreal, QC, Canada; ^2^Sainte-Justine University Hospital and Research Centre of Sainte-Justine Mother and Child University Hospital Center, Montreal, QC, Canada; ^3^Psychiatry Department, Faculty of Medicine, University of Montreal, Montréal, QC, Canada; ^4^The Office of Patient-Family-Caregiver Partnership, Sainte-Justine Mother and Child University Hospital Center, Montréal, QC, Canada

**Keywords:** autism, caregivers, Quebec, courtesy stigma, vulnerability

## Abstract

**Introduction:**

Caregivers of autistic persons often face “courtesy stigma,” a phenomenon by which caregivers experience stigma because of their association with a person whose disability may be stigmatized. Understanding the repercussions of this stigma is crucial not only for caregivers’ mental health but also for the quality of care provided to their dependent. This study aimed to explore courtesy stigma among caregivers of autistic persons in Quebec, examining its prevalence and impact in order to identify groups that are particularly susceptible to negative outcomes.

**Methods:**

This study used a cross-sectional online survey methodology employing quota sampling to collect responses from 194 participants. Data were collected using a computer-assisted web interview (CAWI) platform. The impact of courtesy stigma was measured in terms of care burden, mental health, and overall well-being of caregivers.

**Results:**

The findings revealed that caregivers frequently experience rejection, isolation, and work-related challenges. Notably, caregivers’ health was below average with the lowest reported health outcomes in Quebec. The caregivers who are the most vulnerable to negative outcomes included female caregivers, those aged 45 or older, financially strained households, caregivers of children requiring elevated levels of support, caregivers who isolated due to their autistic dependents, and those who experienced stigmatization directed at themselves or their children in the form of rejection.

Interestingly, 60% of respondents reported that the caregiving burden was “not at all” to “somewhat” difficult, raising questions about factors that may mitigate caregiving challenges over time.

**Conclusion:**

Negative outcomes from courtesy stigma vary depending on certain risk factors and individual characteristic. This study underscores the need for targeted public policies and interventions, particularly for those at a higher risk of experiencing the negative effects of courtesy stigma on the burden of care, overall health, and mental health. By tailoring resources and support for these priority groups, we can better address the challenges faced by families of autistic persons.

## 1 Introduction

Autistic persons and their caregivers are vulnerable populations. Approximately one in 66 children and youth is diagnosed with autism spectrum disorder (ASD) in Canada PHA of (2018). Caregivers of autistic persons face courtesy stigma (Gray, 1993; Ali et al., 2012; Kinnear et al., 2016; Mitter et al., 2019; Papadopoulos et al., 2019; Turnock et al., 2022), which can contribute to approximately 31% of the difficulty in raising an autistic child (Kinnear et al., 2016). Stigma negatively impacts both the burden of care and mental health of caregivers of autistic persons (Zhou et al., 2018; Papadopoulos et al., 2019). Stigma refers to a mark of social disapproval, often based on characteristics such as ethnicity, mental health issues, or disability. It places stigmatized individuals within a hierarchy that results in the loss of privilege, status, and power (Stigma, 2009). More specifically, courtesy stigma is a process defined as the outcome of the relationship between the stigmatized person and the one who stigmatizes, with significant implications for the caregivers of the stigmatized individual (Gray, 2002; Bos et al., 2013). Courtesy stigma negatively impacts both the burden of care and the mental health of caregivers of autistic persons (Zhou et al., 2018; Papadopoulos et al., 2019).

To effectively care for autistic persons, it is also necessary to provide care for their caregivers. This is not just to respond to the needs of caregivers but also because if these caregivers experience mental health issues that could affect the quality of care, it increases the risk of developmental delays in the individuals they care for (Osborne et al., 2008) and may even increase the risk of child maltreatment (Chan et al., 2023). However, despite increasing awareness of the stigma faced by caregivers, our understanding of this issue remains insufficient, and there is a lack of effective strategies to prevent and reduce it (Lodder et al., 2020; Turnock et al., 2022). Furthermore, research on the impact of stigma on caregivers who are particularly vulnerable to the negative outcomes of courtesy stigma is limited.

In this context, there is also a lack of consensus regarding the associated definitions and measurement approaches of courtesy stigma in autism. Čolić (n.d.) and Link and Phelan (2001) proposed the following clarifications: (1) Perceived stigma is parents’ beliefs about negative public attitudes towards them as caregivers or their children. (2) Experienced stigma as actual or past experiences of discrimination, including various forms of disrespect, such as reproaches, long looks, derogatory comments, and limited opportunities in social and professional contexts; and (3) anticipated stigma as an expectation of stigma from others, accompanied by negative emotions such as fear and shame (Link and Phelan, 2001; Čolić, n.d.). Finally, (4) affiliate stigma is the internalization of negative public attitudes by individuals closely associated with the primarily stigmatized person, as their caregivers (Link and Phelan, 2001; Gray, 2002; Bos et al., 2013; Čolić, n.d.). For instance, if the public judges the mother of an autistic child based on the child’s disruptive behavior, she may begin to doubt her parenting skills and may feel inferior, internalizing these negative attitudes towards her (Chan and Lam, 2018). Understanding these definitions is crucial for understanding the challenges faced by caregivers of autistic persons and proposing effective measures to address them.

One of the most accepted models for explaining courtesy stigma in autism is that proposed by Kinnear et al. (2016). Inspired by Link and Phelan (2001), they proposed a theoretical model to explain courtesy stigma among parents of autistic children. This model proposes that the public’s misunderstanding of observable differences in the behavior and characteristics of autistic persons leads to perceived stigma by caregivers. This public misinterpretation can result in negative biases and stereotypes towards autistic persons, resulting in discriminatory behaviors, as rejection, towards both autistic persons and their caregivers. These behaviors can cause social isolation among parents of autistic persons, leading even to anticipated or affiliate stigma. The model developed by Kinnear et al. (2016) focuses on the impact of courtesy stigma on the difficulty of raising an autistic child and the overall impact of stigma in caregiving. This model does not aim to identify caregivers who are the most vulnerable to negative outcomes.

In general, the life span trajectory of autistic persons and their caregivers is not the same for everyone (Keating et al., 2019; Fast et al., 2021). Berg et al. (2016) highlighted the increased exposure to adverse childhood experiences (ACE) among autistic children in their cohort. Additionally, Kerns et al. (2017) observed a heightened risk of ACE, such as mental health problems within the family, particularly among autistic children from low-income families. In this context, mediators have been identified that link courtesy stigma with caregivers’ mental health. The risk factors for a more negative impact include single-parent families, caregiver burden, financial burden, feelings of shame, embarrassment, and social isolation (Papadopoulos et al., 2019). Protective factors to counteract this negative effect are high self-esteem (Cantwell et al., 2015), self-compassion, parental confidence in their parenting skills (Lovell and Wetherell, 2018), and social support (Papadopoulos et al., 2019). These findings suggest that certain caregivers are more susceptible to the negative effects of courtesy stigmas. Identifying these subpopulations is crucial for proposing interventions to enhance effectiveness. However, to the best of our knowledge, research identifying the subpopulations most vulnerable to a more negative life span trajectory in autism is currently lacking.

Therefore, the aim of this study was to sequentially: (1) describe the courtesy stigma on caregivers of autistic persons in Quebec, (2) highlight its detrimental impact on their health and the burden of care, and (3) identify groups that are particularly susceptible to these negative outcomes.

## 2 Methods

### 2.1 Design

This study used a cross-sectional web survey. The sample was obtained from a panel of respondents using quota sampling (Peacock et al., 2017). The participants were members of the Léger Marketing (LM) web panel. LM is a Canadian market research company with a comprehensive pan-Canadian web panel that covers over 200,000 households in Quebec. LM recruitment strategies are multifaceted and include random contact by phone and email, advertising on social media, and word-of-mouth or snowball recruitment. LM has an incentive practice for panel participants, offering reward cards through random draws with a maximum value of $20 to the survey participants.

### 2.2 Sample and procedures

To ensure representation of the population, we calculated the number of families with an autistic persons, considering an autism prevalence of 1.2% among the Quebec population (Diallo et al., 2017), a stigma rate of 50% among caregivers (Kinnear et al., 2016), and a population of 8,575,000 residents in Quebec (Girard et al., 2011). The formula used for sample size calculation was: [z2p(1-p)] / e2 / 1 + [z2p(1-p)] / e2*N, (*z* = 1.96, *e* = 7,025%, *p* = 0.5) (Peacock et al., 2017), where *N* = 71,458 is the population size of caregivers of autistic persons in Quebec, probably exposed to courtesy stigma. We assumed that every autistic person, named in this study as autistic dependent, had a caregiver because we lacked information about the proportion of individuals who were autonomous. The target sample size based on this calculation was 194. The inclusion criteria were adults who were parents, caregivers, or family members of autistic persons, fluent in either French or English, residing in Quebec, and provided care for an autistic family member. Individuals who were autistic themselves were excluded.

**Figure 1:**
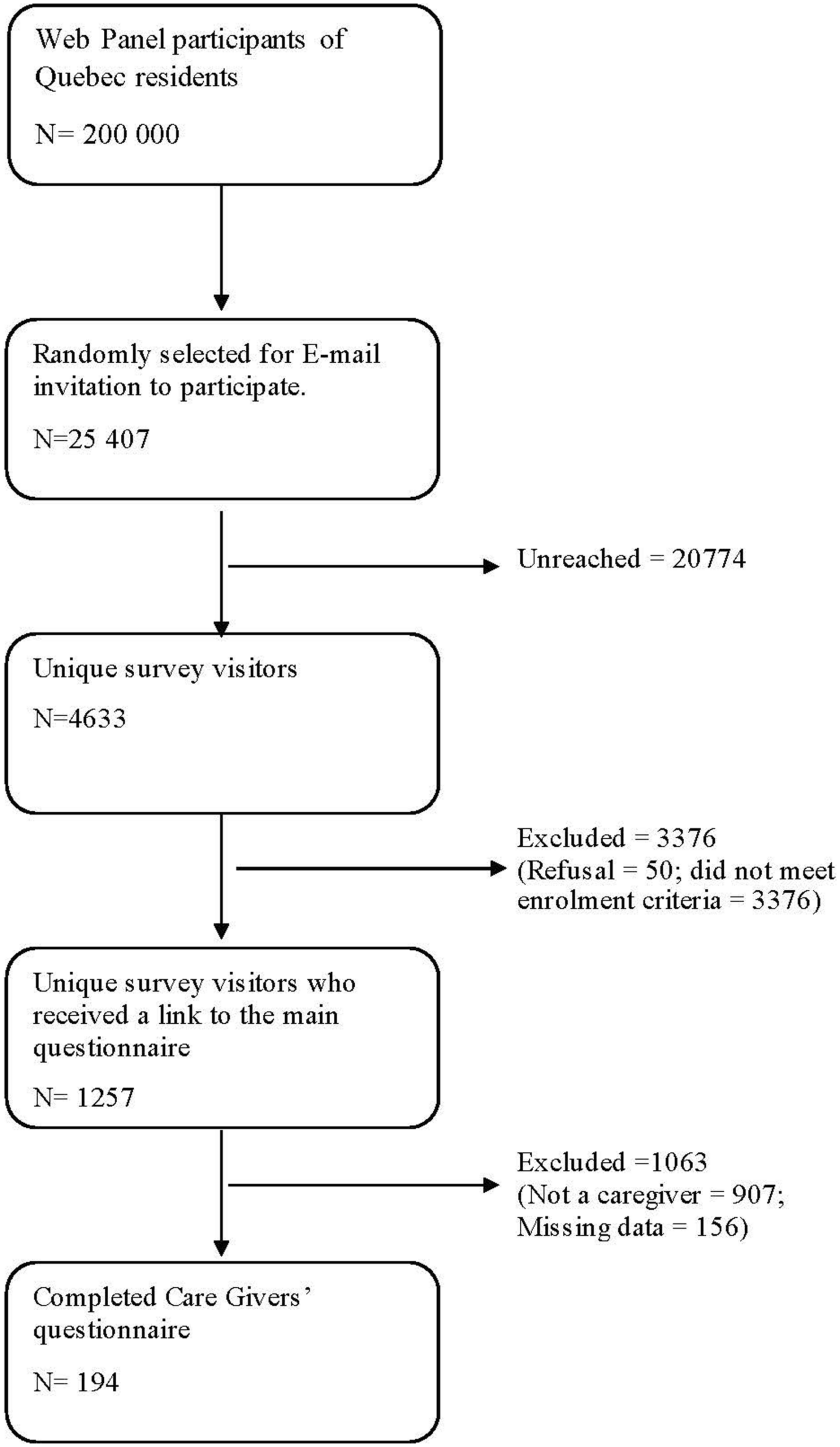
Diagram of participants’ selection.

Recruitment followed the steps illustrated in Figure 1. Email invitations were sent in waves to 25,000 panel members randomly selected from the roster of Quebec residents. The invitations to participate included a unique survey link that could not be shared and could only be used once. As such, panel members who responded to the invitation could access the survey page where they were directed to the selection criteria questions to determine their eligibility to participate in the survey. If participants met all inclusion criteria, they were invited to read and accept the information and consent form and only then were they able to access the survey. The final sample included 194 consenting panelists who self-identified as caregivers of autistic individuals living in their households (Figure 1).

The questionnaire used was initially developed and validated by Kinnear et al. (2016) in a co-production with parents of autistic people to evaluate the impact of courtesy stigma on the caregiving burden of autistic children. This questionnaire was translated into French using a four-step method (René et al., 2011; Bouletreau et al., n.d.). Two bilingual Francophone team members were informed of the study’s objectives and the underlying concepts of the items, and then independently translated the questionnaires into French. Subsequently, the questionnaires were back-translated into the original English language by two bilingual Anglophone individuals who were not informed of the study’s objectives. Finally, a translation committee comprising six bilingual individuals, including field experts and researchers, was formed. Translations and back translations of the original version were compared, and French questionnaires adapted to the Quebec context were proposed through consensus. The final version was reviewed by bilingual and professional French proofreaders. The English and French versions of the questionnaire are presented in the Supplementary materials section.

Data were collected through a Computer-Assisted Web Interviewing (CAWI) interface. A pilot test was conducted with 64 participants, who were not included in the final sample. Data collection took place in July and August 2021. The average survey duration was 13 min. The survey was accessible 24 h a day, 7 days a week, from any computer or portable device (tablets and smartphones) connected to the internet. Reminder emails were sent to invited participants who did not complete the survey.

Data were weighted using 2021 Statistics Canada data (Girard et al., 2011) for age, sex, geographic region, native language, educational attainment, and the proportion of households with an autistic individual to ensure that the sample was representative of the studied population. The weighting details are provided in the Supplementary material. Therefore, based on weighted data, the majority of respondents identified themselves as female (60%), who were under the age of 45 (56%), had a college level of education (58%), and lived with a partner (76%) at the time of the survey. The caregivers supported individuals aged 0–75 years, with a weighted median age of 16.0 years and an interquartile range of 14.2 years. The average time since diagnosis was 5.0 years with an interquartile range of 8.0 years. Moreover, caregivers’ access to social support is reflected in the weighted mean scores obtained for each subscale (out of 100%), including tangible support (mean = 53.99, Standard Deviation =28.3), emotional/informational support (mean = 59.57, Standard Deviation =26.3), positive social interaction (mean = 59.03, Standard Deviation =26.9), and affectionate support (mean = 64.46, Standard Deviation =28.6). Finally, 58% of respondents reported that their autistic child or dependent needed moderate or very important level of support, while 42% stated that only a mild level of support was required (Table 1).

**Table 1:** Unweighted and weighted descriptive statistics for respondents and their autistic dependents.

	Unweighted		Weighted	
**Caregivers’ characteristics**				
Sex - *n* (%)				
Female	110	(56.7)	41	(60.1)
Male	84	(43.3)	27	(39.9)
Age group - *n* (%)				
<45 years	125	(64.4)	43	(62.9)
> = 45 years	69	(35.6)	25	(37.1)
Level of education - *n* (%)				
University	83	(43.0)	14	(21.2)
College	79	(40.9)	38	(57.0)
High School or lower	31	(16.1)	15	(21.8)
Having a partner - *n* (%)				
Yes	121	(62.7)	41	(60.9)
No	72	(37.3)	27	(39.1)
Having difficulties to meet monthly bill payments - *n* (%)				
Not at all	108	(55.7)	35	(51.9)
Slightly	66	(34.0)	25	(37.3)
Extremely	20	(10.3)	7	(10.8)
Questionnaire version - *n* (%)				
French	144	(74.2)	51	(75.0)
English	50	(25.8)	17	(25.0)
Metropolitan region of residence **-** *n* (%)				
Montreal	109	(56.2)	43	(64.0)
Quebec	23	(11.9)	6	(8.6)
Other	62	(32.0)	19	(27.4)
Autistic dependent’s characteristics				
Age of the autistic dependent - Median (Interquartile range)	16.0	(14.0)	16.0	(14.2)
Years since diagnosis - Median (Interquartile range)	6.0	(8.0)	5.0	(8.0)
Level of support the autistic dependent requires - *n* (%)				
Mild level of support	87	(44.8)	29	(42.0)
Important level of support	70	(36.1)	26	(38.4)
Very important level of support	37	(19.1)	13	(19.6)
Total	194	(100.0)	68	(100.0)

### 2.3 Measures

#### 2.3.1 The courtesy stigma in autistic persons’ caregivers

We assigned ratings to the responses and created scores where necessary. The assigned values are enclosed in the parentheses. The questionnaire included the following scales.

##### 2.3.1.1 The caregivers’ perceived Stigma

*Autism-related behaviors scale* assessed the frequency of seven specific behaviors associated with autistic traits, such as head banging, difficulties in making eye contact, and issues with bladder or bowel control. Respondents reported whether their autistic dependents exhibited these behaviors often (3), sometimes (2), rarely (1), or never (0)during the past 6 months. The total score ranged from zero to 21, with higher scores indicating a more frequent occurrence of any of the listed behaviors (α = 0.69).

*Caregivers’ perception of public stereotypes* assessed their perceptions of public stereotypes about individuals on the autism spectrum in two main areas: competence in social roles and causes and characteristics of autism. The first area included a 3-item scale that assessed caregivers’ perceptions of the public’s stereotypes about whether autistic people were unable to hold down a job, live independently, or get married. (*α* = 0.84). The second area used a 5-item scale to evaluate caregivers’ perceptions of public stereotypes such as “Autistic persons cannot be good friends because of their autism,” “Parents can cause autism in their children due to their parenting style,” or “people are mentally ill.” Respondents rated these items on a 3-point scale [most (2), some people (1), or few people (0]) (*α* = 0.62). Scores ranged from zero to 6 and zero to 10 for the two areas, respectively, with higher scores indicating a more frequent occurrence of these perceptions.

Parents were also asked a general question about their perceptions of stigma prevalence. This question was, “Do you think autistic persons are stigmatized?” Caregivers were presented with the following response options: definitely yes (3), probably yes (2), probably no (1), or definitely no (0).

##### 2.3.1.2 The caregivers’ experienced stigma

*Frequency of rejection of autistic dependent by peers* in the last 6 months. Caregivers reported the frequency of seven types of peer rejection behaviors that their dependents faced (often [3], sometimes [2], rarely [1], never [0]). These behaviors included teasing, exclusion from activities, physical bullying, avoidance, hurtful name-calling, perceived as strange, and difficulty forming friendships. The total score was calculated as the sum of item-wise ratings, and ranged from zero to 21, indicating increasing levels of rejection and frequency of exclusion by friends and family (*α* = 0.81).

*Isolation from friends and family* caregivers were asked how often in the past 6 months they decided not to spend time with friends and family because of their autistic dependent behaviors, with the same response options (often [3], sometimes [2], rarely [1], never [0]).

##### 2.3.1.3 The caregivers’ anticipated stigma

*Exclusion by Friends and Family*. Respondents were asked to report how often in the past 6 months (often [3], sometimes [2], rarely [1], never [0]) they felt that themselves and their families were excluded because of their autistic dependent behaviors, with the same response options (often [3], sometimes [2], rarely [1], never [0]).

#### 2.3.2 The overall impact of courtesy stigma on caregivers of autistic persons

Overall assessments of the difficulty of stigma among caregivers and the overall difficulty of caring for an autistic dependent were conducted. We asked the following questions: How difficult has the stigma that is often associated with autism been for you and your family? How difficult has it been for your family to have a child on the autism spectrum? Participants could choose from a scale ranging from (extremely difficult) (5) to “not at all difficult” (1) for these variables.

Caregivers’ overall and mental health statuses were assessed using a five-point Likert scale with five levels: excellent, very good, good, fair, and bad. These questions, in French and English, were sourced from Canadian Community Health Surveys (Government of Canada, 2016). The perceived overall health of an individual is known to have a significant and independent association with various health-related factors, including the presence of specific health issues, utilization of healthcare services, changes in functional status, recovery from health issues, and even mortality (Bowling, 2005). Perceived mental health is strongly associated with social status, social support, a sense of community belonging, and the ability to function in society. Individuals with low perceptions of mental health are more likely to use healthcare services (Fleishman and Zuvekas, 2007).

Participants were asked whether they had reduced their work hours (yes or no) because of caregiving of their autistic dependent.

#### 2.3.3 Identifying higher-risk populations

##### 2.3.3.1 Caregivers’ social support

We used the 19-item Medical Outcome Study Social Support Survey (MOS-SSS) scale (Sherbourne and Stewart, 1991) to assess caregivers’ access to social support. Respondents rated the level of support available to them from one (never) to five (most of the time) when needed. We computed subscale scores for tangible support (four items), emotional/informational support (eight items), affectionate support (three items), and positive social interaction (three items). The transformed score was calculated using the following formula: Transformed Score = (observed score - minimum possible score) / (maximum possible score - minimum possible score) × 100. A high transformed score indicated a high level of perceived social support (Khuong et al., 2018). The internal consistency of both the French and English versions of the scale is α > 0.90 (Robitaille et al., 2011).

##### 2.3.3.2 Having difficulties to meet monthly bill payments

There were three response options: “very or extremely difficult,” “slightly or somewhat difficult,” and “not difficult at all.” This question has been shown to provide relevant information while collecting fewer missing values than traditional questions on income and assets (Hanmer and Cherepanov, 2016).

The sociodemographic variables collected were caregiver age, sex, education level, marital status, place of birth, place of residence, language spoken at home, and language used to answer the questionnaire. We also collected data on the autistic dependent level of support needed according to the DSM-5 as important, moderate, or mild level of support and the time after diagnosis of the supported autistic person.

### 2.4 Analysis

Data were analyzed using IBM SPSS Statistics software (version 25). Data were weighted using the 2021 Statistics Canada data (Girard et al., 2011), as described above. Weighted frequencies were generated and calculated according to several indicators across different demographic groups to determine the frequency of courtesy stigma (Objective 1).

We then assessed four dependent variables (level of difficulty of caring for an autistic dependent, level of impact of stigma on caregivers, caregivers’ mental health status and caregivers’ overall health status) and independent or possible mediating variables such as sex, age group, education level, country of birth, marital status, having difficulties to meet monthly bill payments, language version of the questionnaire (English or French), and level of support required by the autistic dependent. The Kruskal–Wallis test was employed for this purpose because the majority of these variables were on an ordinal scale and the data were not normally distributed.

Bivariate analyses were performed. Spearman’s rank correlation was used to evaluate the relationship between the four dependent variables and potential explanatory variables: frequency of rejection of autistic dependent by peers, frequency of isolation from friends and family, frequency of feeling excluded by family and others, loss in work hours, having difficulties to meet monthly bill payments, level of required care, and access to social support.

Finally, a logistic regression analysis was performed. The four outcome variables were modelled separately (Objectives 2 and 3). To construct the final model, we adhered to the principle of parsimony and included only those explanatory variables that were significantly associated with the outcome, as well as those whose exclusion led to a change in the regression coefficients of other variables by at least 10% (Suresh et al., 2011). We did not apply weights to bivariate and multivariate analyses because the procedures used did not properly handle the data weighting.

This study was approved by the CHU Sainte-Justine Research Ethics Committee (2021–2,853). All the participants provided an online consent form.

## 3 Results

Cronbach’s alpha values for the English and French questionnaires are available in the Supplementary material.

### 3.1 The frequency of courtesy stigma in caregivers of autistic persons

**Figure 2:**
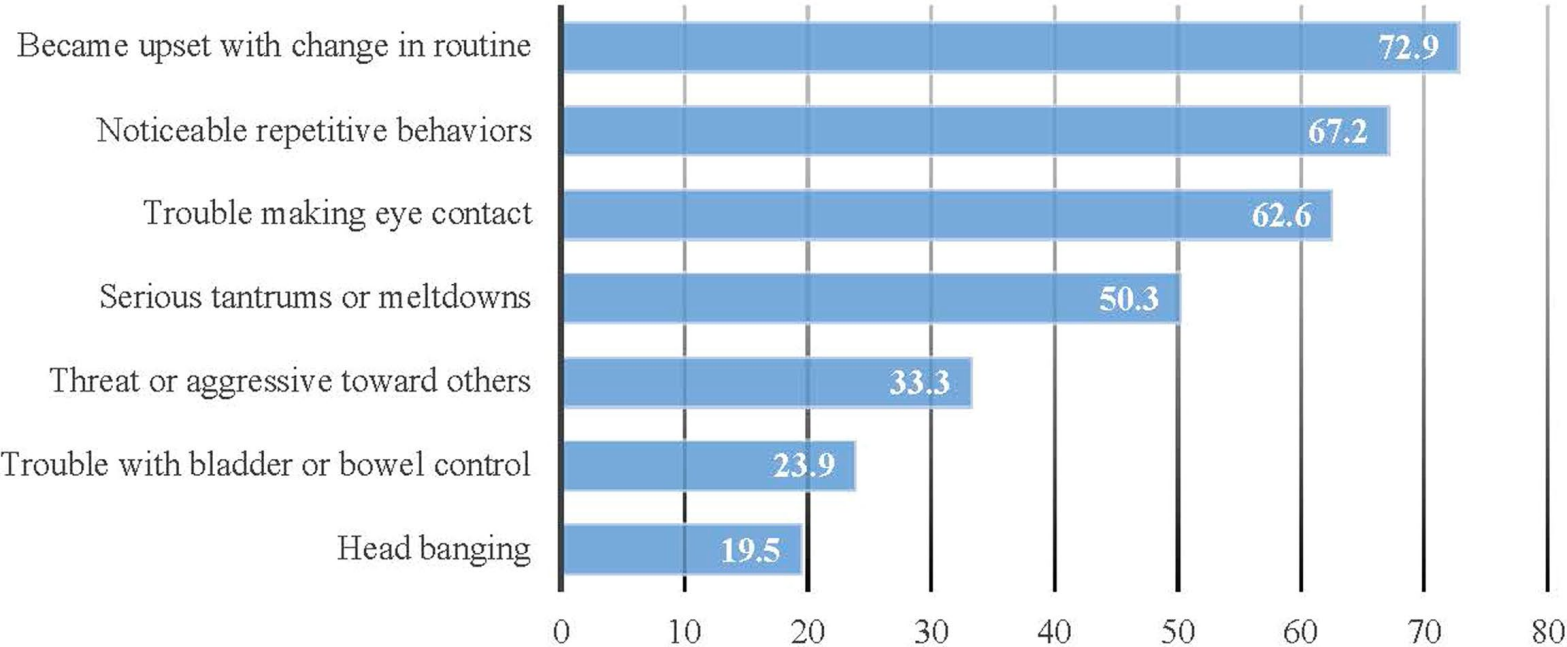
Weighted proportions of respondents reporting that their dependents sometimes or often showed autism-related behaviors during the past six months (Unweighted *N* = 194).

**Figure 3:**
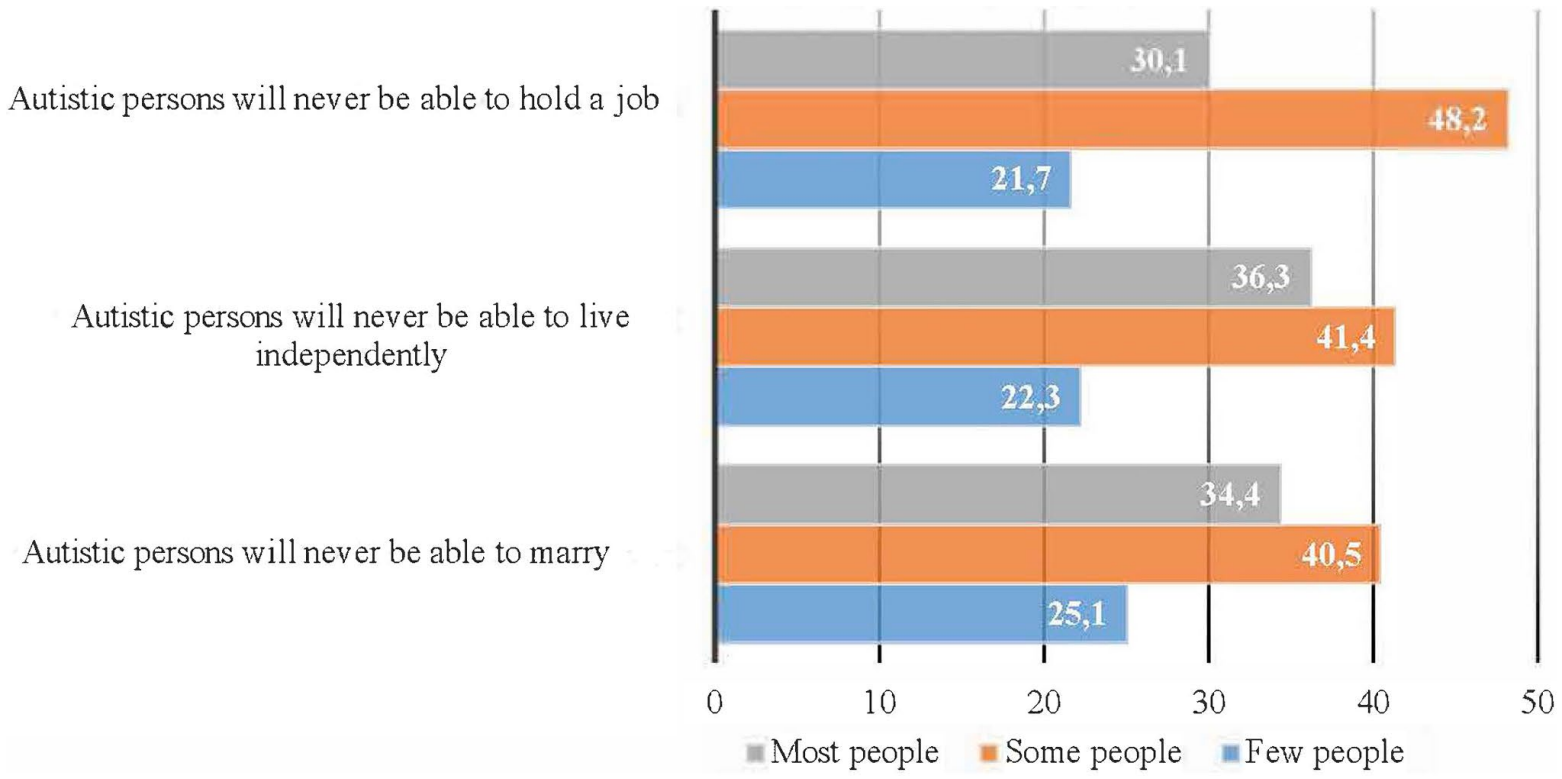
Caregivers’ perceptions of public stereotypes about competencies in the social roles of autistic people; weighted proportions.

**Figure 4:**
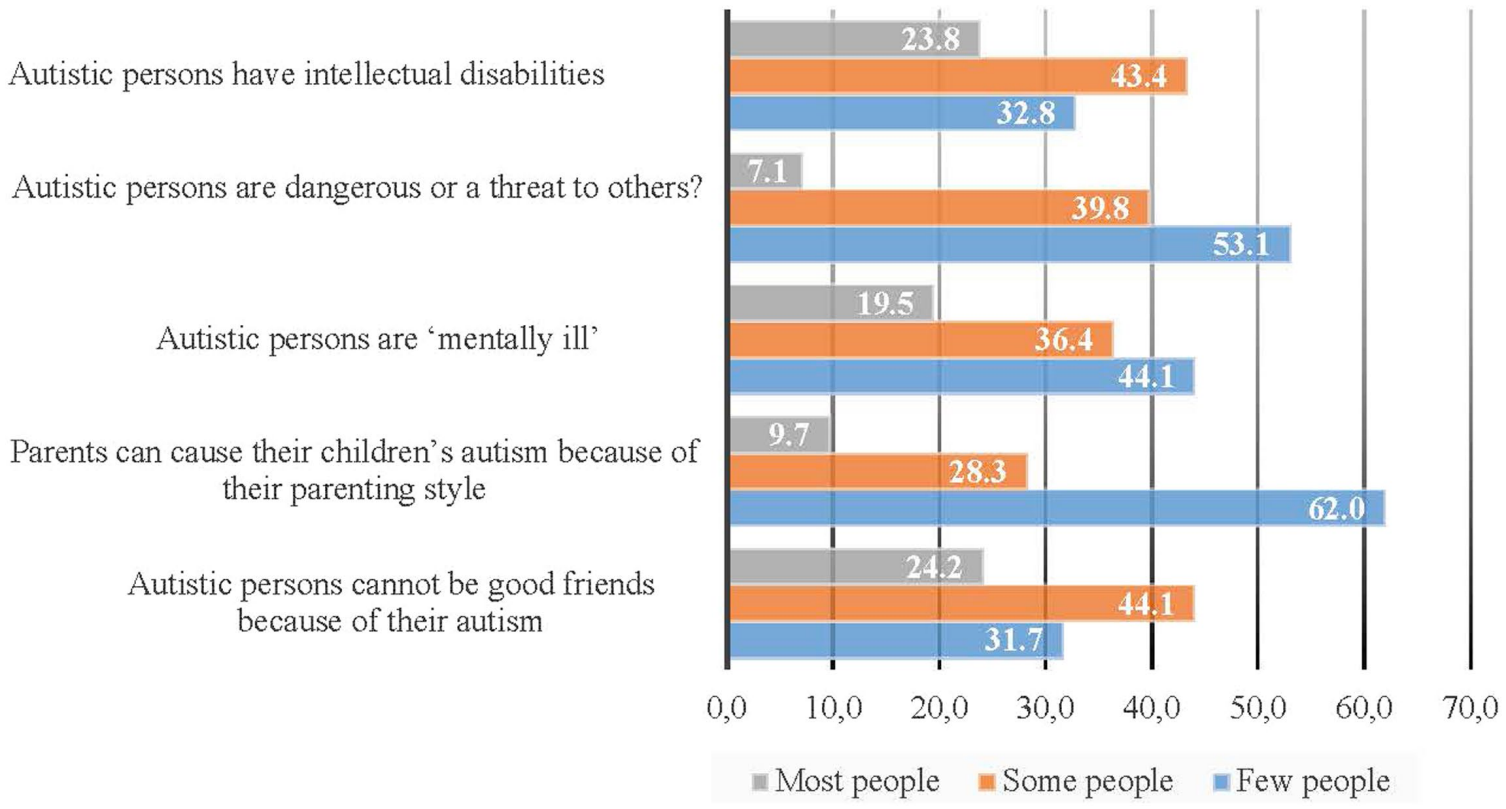
Caregivers’ perceptions of public stereotypes about the causes and characteristics of autism: weighted proportions.

According to the Autism-related Behaviors Scale, the three most frequently observed behaviors in autistic dependents included becoming upset with changes in routine, notable repetitive behaviors, and difficulty in making eye contact (Figure 2). Caregivers perceived that most people and some others held stereotyped beliefs about the social competencies of autistic persons, with over 75% of respondents indicating that autistic persons cannot hold a job, live independently, or marry (Figure 3). Additionally, caregivers perceived that most people and some people hold stereotyped beliefs about the causes and characteristics of autism, with 68.3% feeling that autistic persons cannot be good friends due to their autism, 67.2% feeling that autistic persons have intellectual disabilities, and 55.9% feeling that autistic persons are “mentally ill.” when asked: are autistic persons stigmatized?, 36.7 and 41.9% of caregivers answered, “definitely yes” and “probably yes” respectively, while 14.0% answered “probably not” and only the remaining 7.4% said, “definitely not” (Figure 4).

Caregivers of autistic dependents have experienced courtesy stigma, as demonstrated by the estimate that 46% of autistic dependents have faced rejection by peers. Table 2 provides further information on the frequency of these rejections, including difficulties making friends (64.4%), being avoided by others (50.7%), and being perceived as strange or odd (49.0%). Physical bullying (27%) and being called hurtful names (23.2%) were the least common forms of rejection reported (Table 2).

**Table 2:** Frequency of rejection of autistic dependent by peers (raw *N* = 194; weighted *N* = 68).

	Never %	Rarely %	Sometimes / Often %
How often during the past 6 months your child / [dependent]			
Was teased or called an insulting name	44.2	25.1	30.7
Was left out of activities by other children [peers]	34.5	26.6	38.8
Was physically bullied by other children [peers]	53.0	20.0	27.0
Avoided contact by other children [peers]	26.3	23.0	50.7
Heard child [dependent] called hurtful names or words	47.6	29.2	23.2
Was regarded as weird or odd by other children [peers]	23.4	27.7	49.0
Had difficulty making friends	22.2	13.3	64.4

As show in Table 3, 36% of caregivers reported that they and their families were excluded from social events and activities. Furthermore, half of the caregivers reported that they often or sometimes avoided spending time with friends and family members. Even more, most caregivers (39.3%) found extremely or very difficult to care for an autistic dependent, while 25.5% found it somewhat difficult. Only a small proportion (19.7%) found it a little difficult, and 15.4% found it not at all difficult. Regarding the impact of stigma in their lives, 31.1% of caregivers reported stigma has been extremely or very difficult for them and their families, whereas 23.1% found it somewhat difficult. Only 25.2% found it a little difficult, and 20.6% reported that it did not affect them at all. In terms of caregivers’ self-perceived health, 15% reported fair or bad overall health and 20% reported fair or bad mental health. Finally, 35.6% of respondents reported reducing their work hours because of their autistic dependent (Table 3).

**Table 3:** Unweighted and weighted frequencies of indicators describing caregivers’ courtesy stigma and perceived health status.

	Unweighted *N* = 194		Weighted (*N* = 68)	
	*n*	(%)	*n*	(%)
To what extend individuals autistic persons are stigmatized.				
Certainly not (0)	10	(5.2)	5	(7.4)
Probably not (1)	24	(12.4)	10	(14.0)
Probably yes (2)	91	(46.9)	28	(41.9)
Certainly yes (3)	69	(35.6)	25	(36.7)
How often decided not to spend time with friends and family in the past 6 months				
Never (0)	57	(29.4)	20	(29.5)
Rarely (1)	40	(20.6)	14	(20.5)
Sometimes (2)	62	(32.0)	22	(32.6)
Often (3)	35	(18.0)	12	(17.4)
How often felt excluded along with family in the past 6 months				
Never (0)	79	(40.7)	26	(38.8)
Rarely (1)	49	(25.3)	17	(25.2)
Sometimes (2)	41	(21.1)	16	(23.0)
Often (3)	25	(12.9)	9	(13.0)
How often had to cut back on work hours because of child’s/dependent’s autism in the past 6 months				
Never (0)	83	(42.8)	31	(45.0)
Rarely (1)	42	(21.6)	14	(20.4)
Sometimes (2)	46	(23.7)	16	(24.2)
Often (3)	23	(11.9)	7	(10.4)
How difficult it has been to have a child/dependent on the autism spectrum				
Not at all (0)	29	(14.9)	10	(15.4)
A little (1)	36	(18.6)	13	(19.7)
Somewhat (2)	51	(26.3)	17	(25.5)
Very (3)	46	(23.7)	15	(21.7)
Extremely (4)	32	(16.5)	12	(17.6)
How difficult has the stigma been for the respondent and family.				
Not at all (0)	40	(20.6)	14	(20.6)
A little (1)	46	(23.7)	17	(25.2)
Somewhat (2)	49	(25.3)	16	(23.1)
Very (3)	41	(21.1)	14	(21.2)
Extremely (4)	18	(9.3)	7	(9.9)
Perceived mental health status				
Excellent (0)	21	(10.8)	8	(11.3)
Very good (1)	65	(33.5)	23	(33.6)
Good (2)	71	(36.6)	24	(34.6)
Fair (3)	27	(13.9)	11	(16.7)
Bad (4)	10	(5.2)	3	(3.9)
Perceived overall health status				
Excellent (0)	21	(10.8)	7	(9.9)
Very good (1)	62	(32.0)	20	(29.6)
Good (2)	85	(43.8)	31	(45.8)
Fair (3)	17	(8.8)	7	(9.6)
Bad (4)	9	(4.6)	3	(5.1)

### 3.2 Distribution of variables according to sociodemographic characteristics

The Kruskal-Wallis test results showed that caregivers’ courtesy stigma varied according to their socioeconomic characteristics and the level of support required by their autistic dependent. As shown in Table 4, female respondents were more likely to rate the frequency of dependent autism-related behaviors in the past 6 months than male respondents (H value =10.06, df = 1, *p* = 0.002). Additionally, respondents under the age of 45 tended to rate higher in caregiver’s perception of public stereotypes about competencies in the social roles of autistic people (H value =4.58, df = 1, *p* = 0.032) and caregivers’ perception of public stereotypes about the causes and characteristics of autism (H value =4.18, df = 1, *p* = 0.041) than older respondents.

**Table 4:** Results of the Kruskal-Wallis test examining differences in caregivers’ courtesy stigma indicators according to socioeconomic characteristics and the level of support required by autistic dependents.

		Frequency of child / [dependent] autism-related behavior, past 6 months	Caregivers’ perceptions of public stereotypes about competencies in the social roles of autistic people	Caregivers’ perceptions of public stereotypes about causes and characteristics of autism	Frequency of rejection of child / [dependent] by peers, past 6 months
	*N*	Mean Rank	Mean Rank	Mean Rank	Mean Rank
Sex of respondent					
Female	110	102.70	104.85	104.64	102.94
Male	84	90.69	87.88	88.15	90.38
Chi-Square [H value (df) p-value]		[10.06 (1) *p* = **0.002]**	[4.58 (1) *p* = **0.032**]	[4.18 (1) *p* = **0.041**]	[2.40 (1) *p* = 0.122]
Age group of respondent					
<45 years	125	99.07	91.82	103.92	96.20
> = 45 years	69	94.66	107.80	85.88	99.85
Chi-Square [H value (df) p-value]		[0.71 (1) *p* = 0.399]	[3.79 (1) *p* = 0.052]	[4.67 (1) *p* = **0.031**]	[0.19 (1) *p* = 0.665]
Questionnaire version					
French	144	95.95	98.70	96.39	99.99
English	50	101.96	94.04	100.71	90.34
Chi-Square [H value (df) p-value]		[0.00 (1) *p* = 0.977]	[0.27 (1) *p* = 0.604]	[0.22 (1) *p* = 0.636]	[1.10 (1) *p* = 0.294]
Respondent’s education level					
University	83	94.11	100.01	106.84	101.91
College	79	98.65	96.23	92.59	91.84
High school or less	31	100.55	90.92	81.89	97.02
Chi-Square [H value (df) p-value]		[1.55 (2) *p* = 0.460]	[0.66 (2) *p* = 0.721]	[5.43 (2) *p* = 0.066]	[1.32 (2) *p* = 0.516]
Respondent in couple					
Yes	121	95.48	93.91	91.87	95.23
No	72	99.56	102.19	105.63	99.97
Chi-Square [H value (df) p-value]		[0.60 (1) *p* = 0.438]	[1.04 (1) *p* = 0.307]	[2.79 (1) *p* = 0.095]	[0.33 (1) *p* = 0.568]
Respondent is born in Canada					
Yes	177	96.58	98.12	97.58	97.43
No	15	95.50	77.33	83.77	85.50
Chi-Square [H value (df) p-value]		[2.10 (1) *p* = 0.147]	[2.04 (1) *p* = 0.153]	[0.87 (1) *p* = 0.351]	[0.64 (1) *p* = 0.424]
Difficulty paying monthly bills					
Yes	86	110.22	103.84	101.69	112.06
No	108	87.37	92.45	94.17	85.91
Chi-Square [H value (df) p-value]		[5.21 (1) *p* = **0.022**]	[2.08 (1) *p* = 0.150]	[0.87 (1) *p* = 0.350]	[10.43 (1) *p* = **0.001**]
Level of support required by dependent					
Occasional	87	69.09	79.83	84.16	81.34
Important / very important	107	120.60	111.87	108.35	110.64
Chi-Square [H value (df) p-value]		[0.75 (1) *p* = 0.387]	[16.45 (1) *p* = **0.000**]	[9.06 (1) *p* = **0.003**]	[13.12 (1) *p* = **0.000**]

Bold= *p* < 0.05.

Respondents who reported having difficulties meeting monthly bill payments were compared with those who did not. Variations, including higher ratings for those who had difficulties, were observed in the following areas: frequency of dependent autism-related behaviors (H value = 5.21, df = 1, *p* = 0.022) and frequency of rejection of child or dependent by peers in the past 6 months (H value = 10.43, df = 1, *p* = 0.001).

Compared with those whose autistic dependent required only a mild level of support, those who reported moderate to very high levels of support level of difficulty of stigma on caregivers and their families scored significantly higher on the scales measuring caregivers’ perceptions that autistic people are stigmatized (H value =40.56, df = 1, *p* < 0.001), caregivers’ perceptions of public stereotypes about competencies in the social roles of autistic persons (H value =16.45, df = 1, *p* < 0.001), caregivers’ perceptions of public stereotypes about the causes and characteristics of autism (H value =9.06, df = 1, *p* = 0.003), and the frequency of rejection of child or dependent by peers (H value =13.12, df = 1, *p* < 0.001) (Table 4).

### 3.3 Correlations results

Table 5 presents data that shows the strength and direction of the associations between courtesy stigma indicators and the four outcome variables: (1) overall difficulty of caring for an autistic dependent, (2) overall assessment of difficulty of stigma in caregivers, (3) caregivers’ overall mental health and (4) caregivers’ overall health status. The strength of the correlation was categorized as follows: ≥0.7 = a strong relationship; 0.4–0.6 = a moderate relationship; ≤0.3 = a weak relationship (Akoglu, 2018). None of the correlations can be considered strong. However, most of the correlations indicated weak to moderate associations, all of which were statistically significant (correlation is significant at the 0.01 level, 2-tailed). The highest correlation values were observed between the level of difficulty of stigma on caregivers and their families and the frequency of rejection of autistic dependent by peers (*r* = 0.54; *p* ≤ 0.01), the frequency of feeling excluded by family and others (*r* = 0.50; *p* ≤ 0.01), and the level of difficulty in caring for an autistic child/dependent (*r* = 0.64; *p* ≤ 0.01).

**Table 5:** Spearman’s rank correlation describing associations between courtesy stigma indicators and the four outcome variables.

	Level of difficulty caring for an autistic child/ dependent	Level of difficulty of stigma in caregivers and their families	Caregiver’s perceived overall mental health status	Caregiver’s perceived overall health status
Frequency of child / dependent autism-related behavior, past 6 months	0.397^**^	0.417^**^	0.128	0.120
Caregivers’ perceptions of public stereotypes about competencies in the social roles of autistic people	0.096	0.244^**^	0.180^*^	0.314^**^
Caregivers’ perceptions of public stereotypes about causes and characteristics of autism	0.232^**^	0.329^**^	0.191^**^	0.166^*^
Frequency of rejection of child / [dependent] by peers, past 6 months	0.361^**^	**0.538** ^**^	0.118	0.082
Frequency of isolation (how often decided not to spend time with friends and family in the past 6 months)	0.346^**^	0.416^**^	0.282^**^	0.159^*^
Frequency of feeling excluded by family and others, past 6-months	0.388^**^	**0.505** ^**^	0.270^**^	0.165^*^
Frequency cut back on work hours because of child’s/dependent’s autism in the past 6 months	0.417^**^	0.413^**^	0.178^*^	0.059
Perception that autistic persons are stigmatized	0.302^**^	0.405^**^	0.256^**^	0.212^**^
Level of support required by the autistic-dependent	0.379^**^	0.347^**^	0.106	0.185^**^
Social support_Tangible support	−0.096	−0.094	−0.073	−0.005
Social support_Emotional/informational support	−0.060	−0.078	−0.090	−0.088
Social support_Positive social interaction	−0.101	−0.111	−0.174^*^	−0.157^*^
Social support_Affectionate support	−0.046	−0.091	−0.105	−0.055
Level of difficulty paying monthly bills	0.054	0.158^*^	0.186^**^	0.241^**^
Level of difficulty caring for an autistic child / [dependent]		**0.643** ^**^	0.167^*^	0.080
Level of difficulty of stigma in caregivers and their families			0.169^*^	0.149^*^

^*^Correlation is significant at the 0.05 level (2-tailed); ^**^Correlation is significant at the 0.01 level (2-tailed). Correlation Strength: ≥0.7 = strong relationship; 0.4–0.6 = Moderate relationship; ≤0.3 = Weak relationship (Akoglu, 2018); Bold= *p* < 0.05.

The data also suggest that perceived overall and mental health ratings were positively but weakly associated with “caregivers” perceptions that autistic persons are stigmatized,” “caregiver’s perceptions of public stereotypes about competencies in the social roles of autistic people,” “caregiver’s perception of public stereotypes on causes and characteristics of autism,” “frequency of isolation from friends and family,” “frequency of feeling excluded by family and others,” and “overall assessment of difficulty of stigma in caregivers.” However, there was no statistically significant relationship between the four social support subscales (tangible, emotional/informational, positive social interaction, and affectionate) and the level of difficulty of stigma in caregivers. Nevertheless, positive social interaction was negatively associated with the two perceived health indicators (*r* = −0.16; *p* ≤ 0.05, self-perceived caregivers’ overall health status; *r* = −0.17; *p*-value ≤0.05, caregivers’ overall mental health status). This indicates that higher ratings of positive social interaction are associated with a better self-perceived overall or mental health status.

On the other hand, reporting more difficulties in paying monthly bills were linked to a more negative overall assessment of the difficulty of stigma among caregivers (*r* = 0.16; *p* ≤ 0.05), as well as with reporting poorer overall and mental health status (*r* = −0.24; *p*-value ≤0.05 for caregivers’ overall health status; *r* = −0.19; *p*-value ≤0.05 for caregivers’ overall mental health status). However, this relationship is weak (Table 5).

### 3.4 Results of regressions

Table 6 shows the results of the multivariate regression analyses to predict the level of difficulty in caring for an autistic dependent and the level of difficulty of stigma in caregivers’ and families’ lives. The likelihood of reporting a higher level of difficulty in caring for an autistic dependent (not at all, a little, or somewhat difficult versus very or extremely difficult) significantly varied based on the level of required support by their autistic dependent, specifically, some or a lot of support compared with occasional support (OR = 3.36; 95% [CI = 1.64, 6.90]), the frequency with which caregivers felt excluded by family and others, either sometimes or often compared with never or rarely (OR = 2.28; 95% [CI = 1.06, 4.90]), and the reduction in work hours due to child’s or dependent’s autism, either sometimes or often compared with never or rarely (OR = 3.12; 95% [CI = 1.48, 6.58]).

**Table 6:** Final models of multivariable logistic regression predicting level of difficulty caring for an autistic dependent and stigma-related difficulties in caregivers’ lives controlling for gender, age, financial difficulties, and the language version of the questionnaire.

	Level of difficulty caring for an autistic dependent (not all, a little or somewhat difficult *VS* very or extremely difficult)			Stigma-related difficulties in caregivers’ lives (not all, a little or somewhat difficult *VS* very or extremely difficult)		
	Odds ratio	95% C.I.	Sig.	Odds ratio	95% C.I.	Sig.
Level of support required by the autistic dependent						
Occasional support	1.00					
Some or a lot of support	**3.36**	**(1.64,6.90)**	**0.00**			
Frequency of the dependent’s autistic behavior in past 6 months (Score)				1.09	(0.99,1.19)	0.06
Caregivers’ perceptions of public stereotypes about causes and characteristics of autism						
Score 0–3				1.00		
Score > 3				**2.40**	**(1.14,5.05)**	**0.02**
Frequency of rejection of the dependent by peers, past 6 months (Score)				**1.17**	**(1.08,1.27)**	**0.00**
Frequency caregiver felt excluded by family and others, past 6-months						
Never or rarely	1.00			1.00		
Sometimes or often	**2.28**	**(1.06,4.90)**	**0.04**	**2.47**	**(1.09,5.58)**	**0.03**
Cut back on work hours due to dependent’s autism						
Never or rarely	1.00					
Sometimes or often	**3.12**	**(1.48,6.58)**	**0.00**			
Respondent’s age group						
<de 45 years	1.00					
≳de 45 years	0.50	(0.24,1.03)	0.06			
Questionnaire version						
French				1.00		
English	0.59	(0.27,1.29)	0.19	0.59	(0.24)	0.25

Bold= *p* < 0.05.

The study also revealed that overall assessment of the difficulty of stigma in caregivers’ lives increased in relation to these three variables. Specifically, caregivers who perceived public stereotypes about causes and characteristics of autism to be above three on a total score, as opposed to below three, were more likely to report difficulties ranging from not at all to very or extremely difficult (OR = 2.40; 95% [CI = 1.14, 5.05]). Caregivers who scored above three on the probability of rejection of dependents by peers in the last 6 months (OR = 1.17; 95% [CI = 1.08, 1.27]) and who felt excluded by family and others sometimes or often also in the last 6 months (OR = 2.47; 95% [CI = 1.09, 5.58]) (Table 6).

The data presented in Table 7 indicate that the caregivers’ overall mental health status, ranging from good to bad and very good to excellent, was more likely to be reported as good to bad among those who perceived that autistic persons were stigmatized (probably or certainly, compared to probably not) and those who sometimes or often chose isolation or decided not to spend time with friends and family in the past 6 months (compared to never or rarely). Additionally, men were less likely than women to report poor mental health status (OR = 0.49, 95% CI = [0.26, 0.92]).

**Table 7:** Final models of multivariable logistic regression predicting the association between caregivers’ perceived health status and some indicators of courtesy stigma, controlling for sex, age, financial difficulties, and language version of the questionnaire.

	Self-perceived mental health status (Very good to excellent *vs* good to bad)			Self-perceived overall health status (Very good to excellent *vs* good to bad)		
	Odds ratio	95% C.I.	Sig.	Odds ratio	95% C.I.	Sig
Perception of autistic persons are stigmatized						
Certainly, or probably not	1.00					
Probably or certainly yes	**3.58**	**(1.50,8.52)**	**0.00**			
Stigma-related difficulties in caregivers’ life						
Not all, a little or somewhat difficult	1.00			1.00		
Very or extremely difficult	0.65	(0.31,1.36)	0.25	0.73	(0.34,1.59)	0.43
Caregivers’ perceptions of public stereotypes about causes and characteristics of autism						
Score 0–3				1.00		
Score > 3				**2.16**	**(1.05,4.47)**	**0.04**
Caregivers’ perceptions of public stereotypes about competence in social roles (Score)	1.10	(0.95,1.28)	0.20			
Frequency caregiver felt excluded by family and others, past 6-months						
Never or rarely	1.00					
Sometimes or often	1.48	(0.60,3.63)	0.39			
Frequency of self-isolation (how often decided not to spend time with friends and family in the past 6 months past 6 months)						
Never or rarely	1.00			1.00		
Sometimes or often	**2.20**	**(1.11,4.36)**	**0.02**	0.81	(0.38,1.75)	0.60
Cut back on work hours due to dependent’s autism						
Never or rarely	1.00					
Sometimes or often	1.64	(0.80,3.39)	0.18			
Difficulties in paying monthly bills						
Not difficult at all				1.00		
Difficult	1.00			**1.92**	**(1.01,3.67)**	**0.05**
Score of social positive interaction	0.99	(0.98,1.00)	0.06	0.99	(0.98,1.00)	0.17
Sex						
Female	1.00					
Male	**0.49**	**(0.26,0.92)**	**0.03**			
Respondent’s age group						
<45 years				1.00		
≳45 years				**2.25**	**(1.14,4.44)**	**0.02**
Questionnaire language version						
French	1.00			1.00		
English	0.70	(0.35,1.42)	0.32	**0.44**	**(0.21,0.89)**	**0.02**

Bold= *p* < 0.05.

The data suggest that self-perceived overall health status was more likely to be rated as good to bad if caregivers’ perceptions of public stereotypes about the causes and characteristics of autism were high (score > 3) compared to low (score 0–3) (OR = 2.16, 95% CI = [1.05, 4.47]). Furthermore, experiencing difficulties in paying monthly bills sometimes or often, compared to never or rarely, was also associated with a higher likelihood of rating self-perceived overall health status as good to bad (OR = 1.92, 95% CI = [1.01, 3.67]). Additionally, caregivers aged 45 years or older were more likely to report poor overall health status compared to those younger than 45 (OR = 2.25, 95% CI = [1.14, 4.44]). Lastly, respondents who completed the questionnaire in English were less likely to report poor mental health status compared to those who completed it in French (OR = 0.44, 95% CI = [0.21, 0.89]).

## 4 Discussion

The aims of this research were: (1) to describe the courtesy stigma on caregivers of autistic persons in Quebec, (2) highlight its detrimental impact on their health and burden of care, and (3) identify groups that are particularly susceptible to these negative outcomes.

### 4.1 Living with courtesy stigma as caregivers of autistic persons is frequent in Quebec

**Figure 5:**
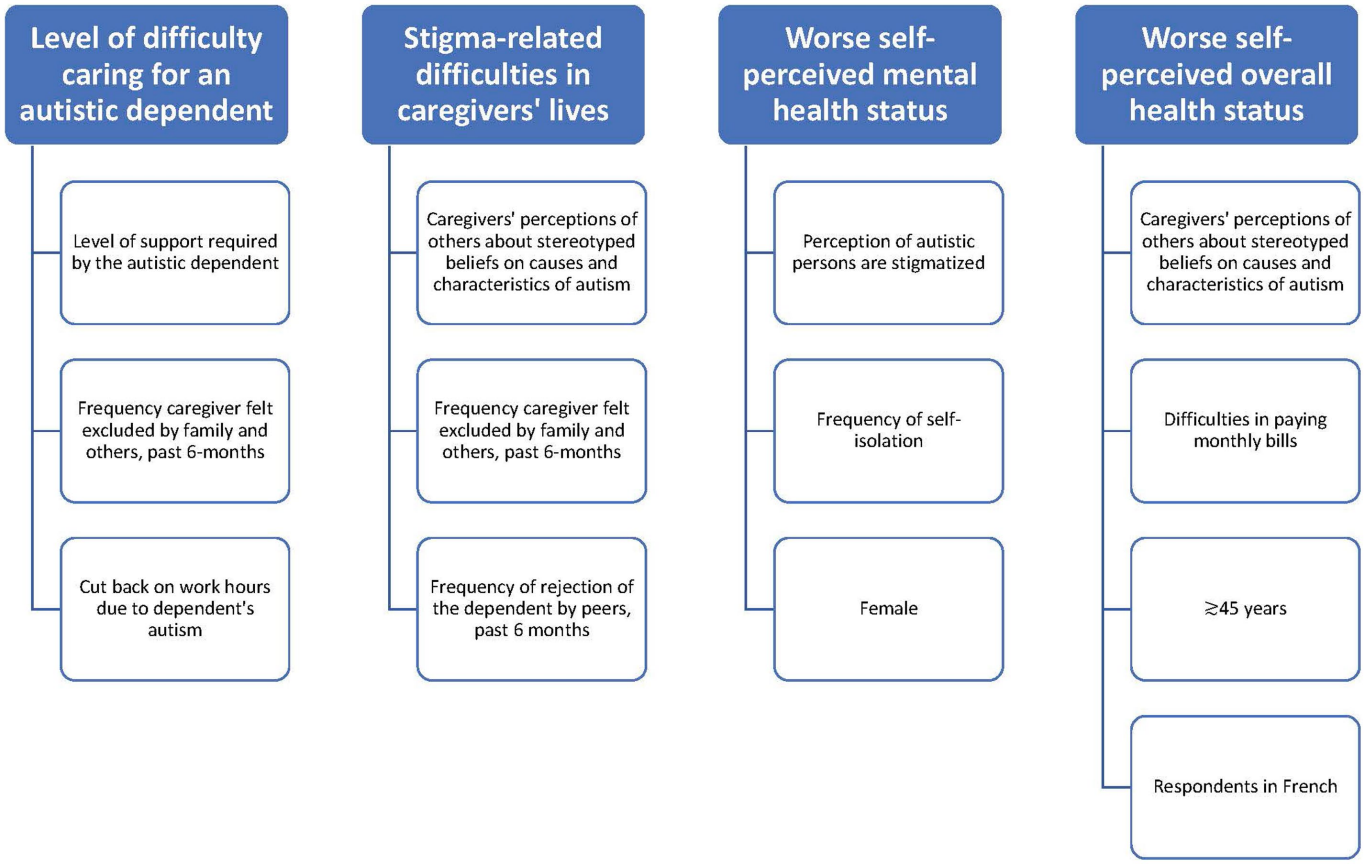
Why does stigma against autistic persons make caregivers’ lives difficult and affect their health.

Our study revealed that courtesy stigma towards caregivers of autistic persons is prevalent in Quebec. Caregivers’ perceptions of public stigma towards the characteristics and causes of autism, as well as the social roles of autistic persons, are common. Our research shows that caregivers frequently experience or witness stigma as discriminatory behaviors towards themselves or their autistic dependents. In addition, caregivers felt excluded by others and often decided not to spend time with their friends and family, isolating themselves from others. The findings of this study, which were derived from a representative panel of respondents recruited from the community, are consistent with those of previous studies (Gray, 1993; Gray, 2002; Kinnear et al., 2016) and recent literature reviews (Ali et al., 2012; Mitter et al., 2019; Papadopoulos et al., 2019; Turnock et al., 2022). This research provides new insights into the courtesy stigma faced by caregivers of autistic persons. Figure 5 summarizes these findings.

### 4.2 Why does the stigma against autistic persons make caregivers’ lives difficult and can affect their health?

#### 4.2.1 Experience of exclusion and rejection as a contributor to the burden of care

Our findings partially diverge from those of Kinnear et al. (2016) in several aspects. In their study, Kinnear et al. (2016) sampled the parents of young children who had just been diagnosed. They recruited participants from a hospital setting and found that stigma accounted for 31% of the challenges in raising an autistic children. Our sample was recruited from the community and from a panel of respondents, indicating that the individuals being cared for were likely older, and had received their diagnosis for a longer period. Thus, in our sample of these characteristics, we found that the stigma-related difficulties in the daily lives of caregivers and their families, even if described by 31% of caregivers as very or extremely difficult, were not in the final regression model explaining the difficulty of caring for an autistic person. These two variables were significantly correlated, and this association had the highest correlation score. However, this variable was excluded because we did not have sufficient statistical power to fit the stable model. Therefore, compared with Kinnear et al. (2), our data may indicate that the stigmatization process continues to be present in the lives of caregivers, but its impact may diminish over time. This was also highlighted by an ethnographic study over 10 years by Gray (2002), which stated that the impact of stigma declined over time. On the other hand, Barker et al. (2011) also proposed that caregivers’ mental health improves over time. However, other forms of stigma are also prevalent.

In fact, research on courtesy stigma related to caregiving for autistic individuals has traditionally focused on affiliate stigma and its impact, mainly on caregivers’ mental health (Gray, 1993; Cantwell et al., 2015; Mitter et al., 2019; Papadopoulos et al., 2019). Interestingly, courtesy stigma can have a negative impact on the burden of care, even without caregivers internalizing it. Our study uncovered an additional factor within the challenges of caring for autistic individuals: caregivers’ experience of exclusion from family activities. This factor is also associated with stigma difficulties in caregivers’ lives as well as their autistic dependent experiences of rejection by peers. Therefore, it is not merely an affiliate stigma that contributes to the burden of care; exclusion and rejection seem to play significant roles, especially over time. Finally, similar to other studies, we also found that the level of support required by their dependents (Liao et al., 2019) and the reduction in hours due to caregiving contribute to the burden of care (Des Rivières-Pigeon and Courcy, 2017). We discuss the implications of perceived stigma below.

#### 4.2.2 The overall health and mental health of caregivers of autistic persons are considerably worse than that of the Quebec population

In our study, caregivers were in poorer health than the general population in Quebec based on self-rated health status indicators. In fact, self-rated health is a self-assessment of an individual’s health and serves as a reliable indicator of their overall well-being. Research indicates that lower self-rated overall health scores are associated with functional decline and increased morbidity (Jylhä, 2009). Estimates from Statistics Canada show that in the year 2021, over 60% of Quebecers aged 12 and older reported that their general health (61.2%) or their mental health (65.8%) was very good or excellent (Government of Canada SC, 2017). Given the differences in the age range of the samples, our estimates (for ages 18 years and older) cannot be optimally compared with those of Statistic Canada (for ages 12 years and above). However, it is notable that the health statistics in our specific sample of caregivers of autistic persons were lower than those of the subgroups with the lowest health statistics estimates in Statistics Canada. In our study, 44.9% of caregivers rated their general health as very good or excellent, compared to the lowest of 53.6% reported by Statistics Canada among 65 years and older Quebecers. Likewise, 39.5% of our caregivers reported very good to excellent mental health status compared to the lowest of 59.5% reported among the 18–34 years old in Statistics Canada. Although we were unable to find significant associations in the stratification analysis, the differences in the means were significant. Therefore, it is imperative to recognize that the mental and overall health of caregivers is a critical variable in ensuring effective support of autistic persons and that the health needs of caregivers of autistic persons must be addressed (Osborne et al., 2008; Chan et al., 2023).

### 4.3 Impact of courtesy stigma may be worsened when other vulnerability factors are present

Research has shown that caregivers without partners are more likely to experience affiliate stigma (Lovell and Wetherell, 2018). According to Kerns et al. (2017), all caregivers face financial constraints, and caregivers who face financial burden and courtesy stigma are particularly vulnerable to social isolation (Dababnah and Parish, 2013). In our study, we found that 34% of caregivers had to reduce their working hours because of their caregiving responsibilities, which may have affected their finances. Research has shown that gender disparities exist in caregiving for autistic persons, with women typically shouldering most of the caregiving burden (Dababnah and Parish, 2013). Our observations show that caregivers who isolated themselves from social activities, caregivers aged 45 years or older, women, and those who perceived that autistic people were stigmatized reported poor mental health more frequently. Moreover, caregivers facing bill payment difficulties and emphasizing others’ perceptions of stigma often reported lower overall health. If our study contained data from families that had had some years since diagnosis, prolonged exposure to stigma over time could eventually have a negative impact on overall and mental health when other vulnerability factors are present.

### 4.4 Inequal impact of courtesy stigma on caregivers

In our sample, 60% of respondents showed they had found it challenging to care for an autistic child or dependent, with responses ranging from “not at all” to “somewhat” difficult. Families dealing with chronic situations have acquired competencies over time (Dinh and Bonner, 2023). It is likely that this also occurs in these families. Gray (2002) conducted a 10-year ethnographic study and observed that a majority of families successfully adapted to parenthood, while a minority did not, with the challenging behaviors of their children being one of the contributing factors. Gray (2002) also suggested that caregivers form new trusted friendships with people who accept their child’s disabilities.

Two other variables associated with perceived stigma among caregivers included perceptions of public stereotypes regarding the causes and characteristics of autism, and caregivers’ perception that autistic individuals are stigmatized. The scale assessing the first variable measures prevalent myths about autism in the general public. Our study demonstrates how simply perceiving stigma can affect the mental and overall health, as well as the caregiving burden, of caregivers.

According to our data, respondents who used the English questionnaire reported higher levels of overall health than those who used it in French. Stigma in courtesy is a cultural phenomenon. The province of Quebec is the only French-speaking region in North America, with an English-speaking population of 10.4% and a population that speaks languages other than English and French accounting for 7.9% (Gouvernement du Québec, 2021). The observed language-based differences in reporting could be attributed to the fact that the English-speaking community may hold distinct beliefs, attitudes, and support networks in relation to autism, which may contribute to variations in how caregivers face stigma. Further studies are needed to confirm this hypothesis.

Contrary to our expectations, social support did not act significantly as a buffer against courtesy stigma. This could be attributed to the gradual depletion of social support networks over time, given that our sample likely consists of caregivers who have been providing care for an extended period (Benson, 2016). Further studies are required to confirm this hypothesis.

### 4.5 Implications for practice

Courtesy stigma affects caregivers unequally. Identifying at-risk populations to propose targeted strategies for caregivers of autistic individuals is a crucial step in creating population-based programs aimed at improving health, reducing the burden of care, and addressing the consequences of courtesy stigma. Based on our data, these at-risk populations could include female caregivers, caregivers aged 45 years or older, those who face difficulties paying their bills and reducing their work hours because of caregiving responsibilities, caregivers with children requiring a higher level of support, those who self-isolate because of stigma, and those who perceive public stigma and experience stigma directed at themselves or their children in the form of exclusion or rejection.

Instruments should be developed to assess caregiving burden and identify caregivers who are more vulnerable to the impacts of courtesy stigma, as proposed by Chan and Lam (2018). This information will allow us to better understand how caregivers cope with the burden of caregiving and courtesy stigma, enabling us to focus our efforts on caregivers who are at greater risk. The potential components of a program to support caregivers of autistic persons include measures to counteract social isolation and provide financial support for those in need.

### 4.6 Implications for research

The persistent presence of courtesy stigma in caregivers’ daily lives raises questions regarding how they navigate and adapt to it. Caregivers may choose to manage, evade, overlook, or endure stigma. It is necessary to consider the concept of lifespan trajectories in caregivers in general (Elder, 1998; Keating et al., 2019; Fast et al., 2021), as well as in caregivers of autistic persons. Given the lifelong nature of autism, and even as autistic persons achieve increasing levels of autonomy, caregivers remain involved for an extended period, often throughout their lifetime (Keating et al., 2019; Fast et al., 2021). This concept has not yet been explored in research on caregiving for autistic persons. Therefore, it is crucial to implement research and targeted interventions throughout the lifespan of families.

Is worth noting that there is currently no comprehensive conceptual model that adequately addresses the complexity of courtesy stigma among caregivers of autistic persons. The burden of care for autistic persons is not always directly related to courtesy stigma, and caregivers’ mental health is not always the result of internalized (affiliation) stigma. A theoretical model that considers the various protective and risk factors for health, burden of caregiving, quality of life, and cultural context across caregivers’ lifespan trajectories is yet to be proposed.

### 4.7 Limits

Autistic people and their families are considered hard-to-reach populations, may possess a higher level of representativeness than other forms of non-probabilistic sampling owing to its origin from a panel of respondents. Panels can be designed to be more representative of the general population than non-probabilistic samples, and responder panels often aim to include a diverse range of demographic groups, which can diminish potential biases compared to non-probabilistic samples. In addition, it is often difficult to reach autistic persons and their families. However, participants in respondent panels may be different from those who do not participate, as Internet access is required, and individuals may have different motivations. Thus, the survey targeted only individuals connected to the Internet; seniors, individuals living in remote regions, visible minorities, and low-income individuals may have been underrepresented. Furthermore, 156 individuals were treated as having missing data and we did not have information on the characteristics of those who did not complete the questionnaire. The analysis and interpretation of results should be considered in this context.

It should also be noted that the survey was conducted during the pandemic, which could have affected the caregivers’ health status. However, it may also have reduced courtesy stigma because families were quarantined. Finally, we did not measure affiliate stigma itself. Some of the variables of the stigma courtesy process we observed, such as avoiding spending time with friends and family, and others’ perception of stigma may indicate perceived or anticipated stigma, but also affiliate stigma. More research is needed to distinguish the impact of the different forms of courtesy stigma specified by Chan et al. (2023), such as affiliate stigma and perceived, anticipated, and experienced stigmas.

## 5 Conclusion

This study revealed that courtesy stigma faced by caregivers of autistic persons is prevalent in Quebec. Caregivers frequently experience discriminatory behaviors towards themselves or their autistic dependents. They often avoided social events and isolated themselves. Caregivers in Quebec have poorer health than the general population, with self-rated health serving as a reliable indicator of overall wellbeing. The health statistics of the caregivers in this study were lower than those of the subgroups with the lowest health estimates in Statistics Canada. It is imperative to address the needs of caregivers of autistic persons to ensure effective support for them, as failure to do so can result in developmental delays and decreased quality of care. The mental and overall health of caregivers is a critical variable in ensuring the effective support of autistic people and other types of stigma, such as rejection, may influence caregivers’ well-being and burden of care. Over time, the effects of caregiver stigma gradually diminished.

This study highlighted that certain demographic groups were more susceptible to adverse health outcomes and a disproportionate caregiving burden. The most vulnerable populations include female caregivers, those aged 45 years or older, individuals with difficulties in meeting monthly bill payments and cutting work hours because of caregiving responsibilities, caregivers of children requiring higher levels of support, those who self-isolate because of their autistic dependents, and those perceiving and experiencing stigmatization in the form of rejection directed at themselves or their children. These findings underscore the importance of implementing public policies and interventions to identify priority populations for intervention, particularly those at the highest risk of experiencing the harmful effects of courtesy stigmas. By raising awareness of the challenges faced by families of autistic persons, resources and support can be directed towards those who are most in need.

## Data availability statement

The datasets generated for this study can be found in the Borealis de l’Université de Montréal DATASET Valderrama, Alena, 2023, “Revealing the Most Vulnerable Groups: Courtesy Stigma in Care-givers of Quebecers Autistic persons”, https://borealisdata.ca/dataset.xhtml?persistentId=doi:10.5683/SP3/ELPYGL.

## Ethics statement

The studies involving humans were approved by Comité d'éthique de recherche du centre de recherche du CHU Sainte-Justine. The studies were conducted in accordance with the local legislation and institutional requirements. The participants provided their written informed consent to participate in this study.

## Author contributions

AV: Conceptualization, Funding acquisition, Investigation, Methodology, Project administration, Resources, Supervision, Writing – original draft, Writing – review & editing. BN: Data curation, Formal analysis, Software, Visualization, Writing – review & editing, Methodology. BF: Conceptualization, Methodology, Writing – review & editing, Investigation. LG: Conceptualization, Writing – review & editing. MG: Conceptualization, Writing – review & editing.
