# Supplementary material for: Revealing the Most Vulnerable Groups: Courtesy Stigma in Caregivers of Autistic Persons in Quebec
Source: Front Psychol. 2024 Jul 31;15:1320816. doi: 10.3389/fpsyg.2024.1320816 (PMC11323393; doi:10.3389/fpsyg.2024.1320816)
Supplement: Supplementary file 1 [file Table_1.DOCX]

**Questionnaire Français**

Veuillez remplir les questions suivantes.

[

**Q0QC.** Dans quelle région du Québec demeurez-vous ?

| **Libellé** | **Valeur** | **Attribut** | **Terminaison** |
| --- | --- | --- | --- |
| Bas-Saint-Laurent | 1 |  |  |
| Saguenay-Lac-Saint-Jean | 2 |  |  |
| Capitale-Nationale | 3 |  |  |
| Mauricie | 4 |  |  |
| Estrie | 5 |  |  |
| Montréal | 6 |  |  |
| Outaouais | 7 |  |  |
| Abitibi-Témiscamingue | 8 |  |  |
| Côte-Nord | 9 |  |  |
| Nord-du-Québec | 10 |  |  |
| Gaspésie/Iles-de-la-Madeleine | 11 |  |  |
| Chaudière-Appalaches | 12 |  |  |
| Laval | 13 |  |  |
| Lanaudière | 14 |  |  |
| Laurentides | 15 |  |  |
| Montérégie | 16 |  |  |
| Centre-du-Québec | 17 |  |  |

**À Q0QCG**

***Une série de questions est ensuite insérée ici pour identifier la provenance plus précise des répondants.***

1. Quel âge avez-vous ?

Moins de 18 ans

De 18 à 24 ans

De 25 à 34 ans

De 35 à 44 ans

De 45 à 54 ans

De 55 à 64 ans

De 65 à 74 ans

75 ans ou plus

Je préfère ne pas répondre

[POSER À TOUS]

1. Êtes-vous :

*Note : comme indiqué par Statistique Canada, les Canadiens transgenres, transsexuels et intersexués doivent indiquer le genre (masculin ou féminin) auquel ils s’identifient le plus.*

Un homme

Une femme

Autre

[POSER À TOUS]

1. Quelle est la langue que vous avez apprise en premier lieu à la maison dans votre enfance et que vous comprenez toujours?

Français

Anglais

Autre

Français et anglais

Français et autre

Anglais et autre

Autre et autre

Je préfère ne pas répondre

[POSER À TOUS]

1. A quel niveau se situe la dernière année de scolarité que vous avez terminée?

Primaire (7 ans ou moins)

Secondaire formation générale ou professionnelle (8 à 12 ans)

Collégial (formation préuniversitaire, formation technique, certificats, attestations ou diplômes

de perfectionnement 13-15 ans)

Universitaire certificats et diplômes

Universitaire 1er cycle Baccalauréat (incluant cours classique)

Universitaire 2e cycle Maîtrise

Universitaire 3e cycle Doctorat

Je préfère ne pas répondre

[POSER À TOUS]

1. Considérez-vous que votre résidence principale se trouve :

...en milieu urbain (dans une grande ville)

...en banlieue ou les environs d’une grande ville

...dans une ville de moyenne ou petite taille

...en milieu rural (à la campagne)

Je ne sais pas

[POSER À TOUS] / [MENTIONS MULTIPLES (POSSIBILITÉ DE COCHER 1 ET 2)] / [ORDRE DE LA LISTE : En ordre]

**QF1**. Êtes-vous (ou une autre personne de votre ménage) une personne autiste?

| **Libellé** | **Valeur** | **Attribut** | **Terminaison** |
| --- | --- | --- | --- |
| Oui, moi personnellement | 1 |  | REMERCIER et TERMINER |
| Oui, une autre personne de mon ménage | 2 |  | PASSER À INTROA |
| Non | 3 | X | PASSER À INTROB |
| Je ne sais pas | 8 | X | PASSER À INTROB |
| Je préfère ne pas répondre | 9 | X | RERMERCI et TERMINER |

1. Veuillez indiquer l'âge de la personne autiste que vous soutenez? __________
2. À quand remonte le diagnostic de la personne autiste que vous soutenez ? ________ années
3. Quel est le niveau de soutien dont la personne autiste que vous soutenez a besoin pour fonctionner dans la communauté ?

Nécessite un soutien très important

Nécessite un soutien important

Nécessite un soutien occasionnel

1. En général, diriez-vous que votre santé physique est…?

Excellente

Très bonne

Bonne

Passable

Mauvaise

1. En général, diriez-vous que votre santé mentale est…?

Excellente

Très bonne

Bonne

Passable

Mauvaise

Voici quelques questions se rapportant au soutien vous étant disponible.

Les gens recherchent parfois de la compagnie, de l’assistance ou autres types de soutien chez les autres. À quelle fréquence ces différents types de soutien vous sont-ils disponibles lorsque vous en éprouvez le besoin? (Cochez la case appropriée).

|  | **Jamais** | **Rarement** | **Quelque fois** | **La plupart du temps** | **Toujours** |
| --- | --- | --- | --- | --- | --- |
| 1. Quelqu’un pour vous aider si vous étiez alité(e). |  |  |  |  |  |
| 1. Quelqu’un sur lequel (laquelle) vous pouvez compter lorsque vous avez besoin d’une oreille attentive. |  |  |  |  |  |
| 1. Quelqu’un qui vous donnera de bons conseils en période de crise. |  |  |  |  |  |
| 1. Quelqu’un pour vous accompagner chez le médecin si vous devez vous y rendre. |  |  |  |  |  |
| 1. Quelqu’un qui vous montre de l’amour et de l’affection. |  |  |  |  |  |
| 1. Quelqu’un avec qui passer de bons moments. |  |  |  |  |  |
| 1. Quelqu’un qui vous donnera de l’information pour vous aider à comprendre une situation. |  |  |  |  |  |
| 1. Quelqu’un à qui vous confier et parler de vous-même. |  |  |  |  |  |
| 1. Quelqu’un qui vous serre dans ses bras. |  |  |  |  |  |
| 1. Quelqu’un qui vous accompagne dans vos moments de détente. |  |  |  |  |  |
| 1. Quelqu’un qui vous préparerait vos repas si vous étiez dans l’incapacité de le faire. |  |  |  |  |  |
| 1. Quelqu’un dont les conseils vous sont précieux. |  |  |  |  |  |
| 1. Quelqu’un avec qui faire des activités pour se distraire et oublier ses soucis. |  |  |  |  |  |
| 1. Quelqu’un pour vous aider avec vos tâches journalières si vous étiez indisposé(e). |  |  |  |  |  |
| 1. Quelqu’un avec qui partager vos tracas et vos peurs les plus intimes. |  |  |  |  |  |
| 1. Quelqu’un vers qui se retourner pour obtenir des suggestions dans le but de composer avec vos problèmes personnels. |  |  |  |  |  |
| 1. Quelqu’un avec qui vous partagez vos moments de loisirs. |  |  |  |  |  |
| 1. Quelqu’un qui comprend vos problèmes. |  |  |  |  |  |
| 1. Quelqu’un à aimer et à se faire désirer |  |  |  |  |  |

***Voici quelques questions concernant votre enfant autiste et concernant la parentalité d'un enfant autiste.***

**Veuillez répondre aux questions suivantes au meilleur de vos connaissances :**

| ***Lors des 6 derniers mois, à quelle fréquence avez-vous observé chez votre enfant le comportement suivant :*** | **Souvent** | **Parfois** | **Rarement** | **Jamais** |
| --- | --- | --- | --- | --- |
| 1. Se cogner la tête |  |  |  |  |
| 1. Menaces ou comportements agressifs envers autrui |  |  |  |  |
| 1. Comportements répétitifs visibles |  |  |  |  |
| 1. Difficulté à établir un contact visuel |  |  |  |  |
| 1. Incontinence urinaire ou fécale |  |  |  |  |
| 1. Crises ou désorganisations intenses |  |  |  |  |
| 1. Contrariété à cause d’un changement de routine |  |  |  |  |

| ***Pensez-vous que la plupart, certaines, ou quelques personnes croient que les personnes autistes ne seront jamais capables de:*** | **La plupart** | **Certaines** | **Quelques** |
| --- | --- | --- | --- |
| 1. Garder un emploi |  |  |  |
| 1. Vivre indépendamment |  |  |  |
| 1. Se marier |  |  |  |

| ***Pensez-vous que la plupart, certaines, ou quelques personnes croient que:*** | **La plupart** | **Certaines** | **Quelques** |
| --- | --- | --- | --- |
| 1. Les personnes autistes ne peuvent pas être de bons amis parce qu’elles sont autistes |  |  |  |
| 1. Les parents peuvent causer l’autisme de leur enfant à cause de leur façon d’être parents |  |  |  |
| 1. Les personnes autistes sont “malades mentales” |  |  |  |
| 1. Les personnes autistes sont dangereuses ou sont une menace pour les autres |  |  |  |
| 1. Les personnes autistes ont une déficience intellectuelle |  |  |  |

| ***À quelle fréquence dans les 6 derniers mois votre enfant***: | **Souvent** | **Quelques fois** | **Rarement** | **Jamais** |
| --- | --- | --- | --- | --- |
| 1. A été agacé ou traité d’un nom insultant |  |  |  |  |
| 1. A été exclu d’activités par ses pairs |  |  |  |  |
| 1. A été intimidé physiquement par ses pairs |  |  |  |  |
| 1. A évité le contact avec d’autres enfants |  |  |  |  |
| 1. A entendu des enfants le traiter de noms ou de mots blessants |  |  |  |  |
| 1. A été considéré comme bizarre ou étrange par les autres |  |  |  |  |
| 1. A eu de la difficulté à se faire des amis |  |  |  |  |

| ***À quelle fréquence dans les 6 derniers mois avez vous*** | **Souvent** | **Quelques fois** | **Rarement** | **Jamais** |
| --- | --- | --- | --- | --- |
| 1. Décidé de ne pas passer de temps avec vos amis et votre famille |  |  |  |  |
| 1. Ressenti que vous et votre famille étiez exclus |  |  |  |  |
| 1. Avez-vous dû manquer des heures de travail à cause de l’autisme de votre enfant |  | |  | |

| **Dans quelle mesure:** | **Vraiment beaucoup** | **Beaucoup** | **Plutôt** | **Un peu** | **Pas du tout** |
| --- | --- | --- | --- | --- | --- |
| 1. La stigmatisation souvent associée à l’autisme a-t-elle été difficile pour vous et votre famille? |  |  |  |  |  |
| 1. Le fait d’avoir un enfant appartenant au spectre de l’autisme a-t-il été difficile pour vous et votre famille ? |  |  |  |  |  |

**Dans quelle mesure:**

| 1. Les personnes autistes sont stigmatisées | **Certainement** | **Probablement** | **Probablement pas** | **Certainement pas** |
| --- | --- | --- | --- | --- |

1. Êtes-vous…?

Célibataire

Marié(e) ou conjoint(e) de fait

Veuf (veuve)

Séparé(e)

Divorcé(e)

Je préfère ne pas répondre

1. Êtes-vous né(e) au Canada?

Oui

Non

Je ne sais pas

Je préfère ne pas répondre

| 1. Dans quelle mesure est-il difficile pour vous de payer vos factures mensuelles? | **Pas difficile du tout** | **Légèrement ou plutôt difficile** | **Très ou extrêmement difficile** |
| --- | --- | --- | --- |

1. Qu’est-ce que l’autisme dans vos propres mots?

________________

Merci de votre participation !

Si vous sélectionnez "CONTINUER", les données seront conservées. Si vous décidez de quitter la page, vos données seront supprimées.
