# Supplementary material for: Revealing the Most Vulnerable Groups: Courtesy Stigma in Caregivers of Autistic Persons in Quebec
Source: Front Psychol. 2024 Jul 31;15:1320816. doi: 10.3389/fpsyg.2024.1320816 (PMC11323393; doi:10.3389/fpsyg.2024.1320816)
Supplement: Supplementary file 2 [file Table_2.DOCX]

**Questionnaire anglais**

[POSER À TOUS]

Please answer the following questions.

**Q0QC.** In which region of Quebec do you live?

| **Libellé** | **Valeur** | **Attribut** | **Terminaison** |
| --- | --- | --- | --- |
| Bas-Saint-Laurent | 1 |  |  |
| Saguenay-Lac-Saint-Jean | 2 |  |  |
| Capitale-Nationale | 3 |  |  |
| Mauricie | 4 |  |  |
| Estrie | 5 |  |  |
| Montréal | 6 |  |  |
| Outaouais | 7 |  |  |
| Abitibi-Témiscamingue | 8 |  |  |
| Côte-Nord | 9 |  |  |
| Nord-du-Québec | 10 |  |  |
| Gaspésie/Iles-de-la-Madeleine | 11 |  |  |
| Chaudière-Appalaches | 12 |  |  |
| Laval | 13 |  |  |
| Lanaudière | 14 |  |  |
| Laurentides | 15 |  |  |
| Montérégie | 16 |  |  |
| Centre-du-Québec | 17 |  |  |

**À Q0QCG**

***Une série de questions est ensuite insérée ici pour identifier la provenance plus précise des répondants.***

[POSER À TOUS]

1. How old are you?

Under 18

Between 18 and 24

Between 25 and 34

Between 35 and 44

Between 45 and 54

Between 55 and 64

Between 65 and 74

75 or older

I prefer not to answer

[POSER À TOUS]

1. You are…?

*Note: As indicated by Statistics Canada, transgender, transsexual, and intersex Canadians should indicate the gender (male or female) with which they most associate themselves.*

A man

A woman

Other

[POSER À TOUS]

1. What is the language you first learned at home in your childhood and that you still understand?

French

English

Other

English and French

French and other

English and other

Other and other

I prefer not to answer

[POSER À TOUS]

1. What is the last year of education that you have completed?

Elementary (7 years or less)

High school, general or vocational (8 to 12 years)

College (pre-university, technical training, certificate, accreditation or

advanced diploma (13 to15 years)

University certificates and diplomas

University Bachelor (including classical studies)

University Master's degree

University Doctorate (PhD)

I prefer not to answer

[POSER À TOUS]

1. Do you consider that your main residence is :

...in an urban environment (in a large city)

...in the suburbs or the surroundings of a large city

...in a medium or small town

...in a rural area (in the countryside)

I don't know

[POSER À TOUS] / [MENTIONS MULTIPLES (POSSIBILITÉ DE COCHER 1 ET 2)] / [ORDRE DE LA LISTE : En ordre]

**QF1**. Are you (or anyone else in your household) an autistic person?

| **Libellé** | **Valeur** | **Attribut** | **Terminaison** |
| --- | --- | --- | --- |
| Yes, me personally | 1 |  | REMERCIER et TERMINER |
| Yes, another person in my household | 2 |  | PASSER À INTROA |
| No | 3 | X | PASSER À INTROB |
| I don’t know | 8 | X | PASSER À INTROB |
| I prefer not to answer | 9 | X | RERMERCI et TERMINER |

1. Indicate the age of the autistic person you support: __________
2. When was the diagnosis made for the autistic person you support? _____years ago
3. What is the level of support that the autistic person you support needs to function in the community?

Requires a lot of support

Requires some support

Requires an occasional support

1. In general, would you say that your health is:

Excellent

Very good

Good

Fair

Bad

1. In general, would you say that your mental health is:

Excellent

Very good

Good

Fair

Bad

**People sometimes seek companionship, assistance, or other types of support from others.**

**How often are these different types of support available to you when you need them? (Check the appropriate box)**

|  | **Never** | **Rarely** | **Sometimes** | **Most of the Time** | **Always** |
| --- | --- | --- | --- | --- | --- |
| 1. Someone to help you if you were bedridden |  |  |  |  |  |
| 1. Someone you can count on when you need someone to be there for you. A listening ear |  |  |  |  |  |
| 1. Someone who will give you good advice in times of crisis. |  |  |  |  |  |
| 1. Someone to accompany you to the doctor's office if you need to go. |  |  |  |  |  |
| 1. Someone who shows you love and affection |  |  |  |  |  |
| 1. Someone to spend quality time with |  |  |  |  |  |
| 1. Someone who will give you information to help you understand a situation. |  |  |  |  |  |
| 1. Someone to whom you can confide your problems and talk about yourself. |  |  |  |  |  |
| 1. Someone to give you a hug. |  |  |  |  |  |
| 1. Someone to accompany you in your moments of relaxation. |  |  |  |  |  |
| 1. Someone who would prepare meals for you if you were unable to do so. |  |  |  |  |  |
| 1. Someone whose advice is valuable to you. |  |  |  |  |  |
| 1. Someone to do activities with to distract you, to take your mind off your concerns. |  |  |  |  |  |
| 1. Someone to help you with your daily chores if you are indisposed. |  |  |  |  |  |
| 1. Someone to share your most intimate worries and fears with. |  |  |  |  |  |
| 1. Someone to turn to for suggestions on how to deal with your personal problems. |  |  |  |  |  |
| 1. Someone to share your leisure time with. |  |  |  |  |  |
| 1. Someone who understands your problems. |  |  |  |  |  |
| 1. Someone to love and make you feel wanted. |  |  |  |  |  |

**Here are some questions about your autistic child and about parenting an autistic child**

**Please answer the following questions to the best of your knowledge:**

| ***How often during the past 6 months did your child show the following behavior?*** | **Often** | **Sometimes** | **Rarely** | **Never** |
| --- | --- | --- | --- | --- |
| 1. Head banging |  |  |  |  |
| 1. Threat or aggressive toward others |  |  |  |  |
| 1. Noticeable repetitive behaviors |  |  |  |  |
| 1. Trouble making eye contact |  |  |  |  |
| 1. Trouble with bladder or bowel control |  |  |  |  |
| 1. Serious tantrums or meltdowns |  |  |  |  |
| 1. Became upset with change in routine |  |  |  |  |

| ***Do you think (most/some/few) people believe that individuals with autism will never be able to ____.?*** | **Most** | **Some** | **Few** |
| --- | --- | --- | --- |
| 1. Hold a job |  |  |  |
| 1. Live independently |  |  |  |
| 1. Marry |  |  |  |

| **From your point of view, do most people, some people or only a few people believe that …** | **Most people** | **Some people** | **Few people** |
| --- | --- | --- | --- |
| 1. Individuals with autism cannot be good friends because of their autism |  |  |  |
| 1. Parents can cause their children’s autism because of their parenting style |  |  |  |
| 1. Individuals with autism are ‘mentally ill’ |  |  |  |
| 1. Individuals with autism are dangerous or a threat to others? |  |  |  |
| 1. Individuals with autism have intellectual disabilities |  |  |  |

| ***How often during the past 6 months your child*** | **Often** | **Sometimes** | **Rarely** | **Never** |
| --- | --- | --- | --- | --- |
| 1. Was teased or called an insulting name |  |  |  |  |
| 1. Was left out of activities by other children |  |  |  |  |
| 1. Was physically bullied by other children |  |  |  |  |
| 1. Avoided contact by other children |  |  |  |  |
| 1. Heard child called hurtful names or words |  |  |  |  |
| 1. Was regarded as weird or odd by other children |  |  |  |  |
| 1. Had difficulty making friends |  |  |  |  |

| ***How often during the past 6 months you as parent*** | **Often** | **Sometimes** | **Rarely** | **Never** |
| --- | --- | --- | --- | --- |
| 1. Decided not to spend time with friends and family |  |  |  |  |
| 1. Felt that you and your family were excluded |  |  |  |  |
| 1. Cut back on work hours because of child’s autism |  |  |  |  |

| **How difficult…** | **Extremely** | **Very** | **Somewhat** | **A little** | **Not at all** |
| --- | --- | --- | --- | --- | --- |
| 1. Has the stigma that is often associated with autism been for you and your family? |  |  |  |  |  |
| 1. Has it been for you and your family to have a child on the autism spectrum? |  |  |  |  |  |

| **To what extent…** | **Definitely**  **yes** | **Probably**  **yes** | **Probably**  **no** | **Definitely**  **no** |
| --- | --- | --- | --- | --- |
| 1. Individuals with autism are stigmatized |  |  |  |  |

1. What is your marital status? ~~Are you?~~

Single

Married or living together

Widowed

Separated

Divorced

I prefer not to answer

1. Were you born in Canada?

Yes

No

I prefer not to answer

|  | **Not difficult at all** | **Slightly or somewhat difficult** | **Very or extremely difficult** |
| --- | --- | --- | --- |
| 1. “How difficult is it for you to meet the monthly payments on your bills? |  |  |  |

1. What is autism in your own words?

Thank you for your participation!

If you select "CONTINUE", the data will be retained. If you decide to close the window, your data will be deleted.
