# Supplementary material for: Revealing the Most Vulnerable Groups: Courtesy Stigma in Caregivers of Autistic Persons in Quebec
Source: Front Psychol. 2024 Jul 31;15:1320816. doi: 10.3389/fpsyg.2024.1320816 (PMC11323393; doi:10.3389/fpsyg.2024.1320816)
Supplement: Supplementary file 3 [file Data_Sheet_1.docx]

**Revealing the Most Vulnerable Groups: Courtesy Stigma in Caregivers of Quebecers Autistic persons**

**Suplementary materiels**

Weighting

The results were weighted by age, sex, region, mother tongue, education and the proportion of households with an autistic person to make the sample representative of the entire study population. The weight variable (WEIGHT) is included in the raw data file. The detailed weighting is as follows:

| **No** | **Variable age sex** | **Poids** |
| --- | --- | --- |
| 1 | One man, Other AND From 18 to 24 years | 0.0496 |
| 2 | One man, Other AND From 25 to 34 years | 0.0763 |
| 3 | A man, Other AND From 35 to 44 years | 0.0811 |
| 4 | A man, Other AND From 45 to 54 years | 0.0861 |
| 5 | A man, Other AND From 55 to 64 years | 0.0937 |
| 6 | One man, Other AND From 65 to 74 years,75 years or more | 0.0963 |
| 7 | A woman AND From 18 to 24 years | 0.0528 |
| 8 | A woman AND From 25 to 34 years | 0.0776 |
| 9 | A woman AND From 35 to 44 years | 0.0826 |
| 10 | A woman AND From 45 to 54 | 0.0865 |
| 11 | A woman AND From 55 to 64 | 0.0958 |
| 12 | A woman AND From 65 to 74 years, 75 years or more | 0.1216 |

| **No** | **Variable region** | **Poids** |
| --- | --- | --- |
| 1 | One man, Other AND Mtl RMR | 0.2404 |
| 2 | A man, Other AND Qc CMA | 0.0490 |
| 3 | One man, Other AND East | 0.0402 |
| 4 | A man, Other AND Centre | 0.0721 |
| 5 | One man, Other AND West | 0.0815 |
| 6 | A woman AND Mtl RMR | 0.2578 |
| 7 | A woman AND Qc RMR | 0.0520 |
| 8 | A woman AND Est | 0.0415 |
| 9 | A Woman AND Centre | 0.0764 |
| 10 | A Woman AND West | 0.0892 |

| **No** | **Variable mother tongue** | **Poids** |
| --- | --- | --- |
| 1 | A man, Other AND French | 0.3729 |
| 2 | A man, Other AND English, Other, I prefer not to answer | 0.1102 |
| 3 | A woman AND French | 0.4003 |
| 4 | A woman AND English, Other, I prefer not to answer | 0.1165 |

| **No** | **Variable education** | **Poids** |
| --- | --- | --- |
| 1 | Primary (7 years or less), Secondary general or vocational training (8 to 12 years, College (pre-university training, technical training, I  prefer not to answer | 0.7442 |
| 2 | University certificates and diplomas, University undergraduate Baccalaureate  (including classical course, University 2nd cycle Master’s degree, University 3rd cycle Doctorate | 0.2558 |

| **No** | **Variable autistic person in the family** | **Poids** |
| --- | --- | --- |
| 1 | Yes, another person in my household | 0.0590 |
| 2 | Non | 0.9410 |

**Psychometric characteristics of the used scales - our data**

| Variables | Total   Score | CI | Cronbach |
| --- | --- | --- | --- |
| Child’s autism-related behaviors (α= 0.69) | 18.36 | 17.66-19.07 | 0.836558(F)  0.800584(A) |
| The perception of public stereotypes (α=0.84) | 5.66 | 5.38-5.95 | 0.897657(F)  0.905291(A) |
| Beliefs about the causes and characteristics of autism (α=0.62) | 11.29 | 10.93-11.65 | 0.787850(F)  0.732931(A) |
| The frequency of rejection of the child by peers (α=0.81) | 19.34 | 18.54-20.14 | 0.900649(F)  0.893886(A) |
| The isolation and exclusion of the family and the impact on the working hours of ordinal parents | 8.51 | 8.12-8.89 | 0.836558(F)  0.814979(A) |
| Assessing the difficulty of stigma in the lives of parents and the difficulty of raising an ordinal autistic child | 6.17 | 5.84-6.50 | 0.769268(F)  0.814563(A) |
|  |  |  |  |
|  |  |  |  |

F : French, A : English
